# Dog ownership enhances anchored personal relationships and sense of community: A comparison with incidental interactions and friendships

**DOI:** 10.1371/journal.pone.0336957

**Published:** 2025-12-10

**Authors:** Itaru Ishiguro, Shinnosuke Kodama, Hikari Koyasu, Miho Nagasawa, Takefumi Kikusui

**Affiliations:** 1 College of Contemporary Psychology, Rikkyo University, Niiza, Saitama, Japan; 2 School of Veterinary Medicine, Azabu University, Sagamihara, Kanagawa, Japan; UFSJ: Universidade Federal de Sao Joao del-Rei, BRAZIL

## Abstract

Dogs are known to be catalysts for human-human relationships. However, there is insufficient quantitative research that directly compares dog owners’ human-human relationships with those of non-owners. This study focused on whether dog owners are more likely to have human-human relationships and engage in social interactions within their neighborhoods. A previous model considered incidental interactions—which occur spontaneously between passersby in public settings—and friendships, as human-human relationships fostered by dog ownership. This study also considered anchored personal relationships, which are the types of relationships that dog owners would typically cultivate within their neighborhoods. Anchored personal relationships are relationships highly embedded within a social context and exist solely in a shared time, place, and activity. This study examined the associations between dog ownership and incidental interactions, friendships, and anchored personal relationships. We also examined the potential mediating effects of these relationships on the association between dog ownership and a sense of community. Analyzed data included 377 participants from a social survey conducted in a suburb of the Tokyo metropolitan area. We used generalized structural equation modeling to examine indirect effects. The findings revealed a positive correlation between dog ownership and having anchored personal relationships, as well as between dog ownership and having incidental interactions. However, after controlling for demographics, dog ownership did not increase the likelihood of having friends in one’s neighborhood. All types of relationships were positively correlated with a stronger sense of community. However, only anchored personal relationships mediated the effect of dog ownership on sense of community. These findings support the proposition that anchored personal relationships should be considered alongside incidental interactions and friendships when studying human-human relationships fostered by dog ownership. Furthermore, exploratory analyses revealed that ownership of cats and other pets were not correlated with the relationships or a stronger sense of community.

## Introduction

### Dog ownership and personal relationships

A substantial body of research has investigated the associations between dog ownership and the owners’ physical and mental health. Although their findings have been inconsistent, a considerable number of studies have demonstrated improvements in both physical and mental health (for reviews, see [1,2]). In recent years, dogs have been found to catalyze human-human interactions and relationships [3–5]. The presence of dogs increases the likelihood of being spoken to by acquaintances and strangers [6]. Dog ownership also increases opportunities for contact with neighbors because it requires regular walking [5,7].

There are few quantitative studies focused on the associations between dog ownership and human-human relationships (henceforth, the term personal relationships will be employed in this study). In particular, there have been limited quantitative comparisons of the number and quality of dog owners’ personal relationships and those of nonowners. Several studies have focused on social isolation or loneliness (for reviews of these studies, see [8,9]), neither of which quantify personal relationships. Furthermore, only a few studies have addressed the community-level impact of pet dogs. Wood et al. conducted a relatively large quantitative survey and found that pet owners gained more acquaintances in their neighborhoods [3,5], a greater sense of community [8], and more social capital, such as neighbor relationships characterized by trust and reciprocity [4,7]. Furthermore, these associations were demonstrated to be stronger for dog owners than for owners of other pets, particularly when dog owners regularly walk their dogs [5,7].

According to Wood et al.’s [5] model, pets generate social support by facilitating incidental interactions and the formation of friendships, creating social capital. Having pets is expected to increase the probability of incidental interactions given their capacity to serve as a catalyst for contact with strangers or acquaintances. Friendships sometimes form following these interactions facilitated by the presence of pets. Social capital, from which people draw social support, can be derived from both incidental interactions and friendships. Consequently, Wood et al. [5] argued that pet ownership influences the quantity of social support received, mediated by incidental interactions and friendships.

In addition to incidental interactions and friendships, this study considered personal relationships that fall between these two categories. To capture these relationships, we used the concept of *anchored personal relationships* [9,10]. This concept refers to personal relationships which involve “recurring interaction and interdependencies that develop between individuals over time but are tied to a particular public place and a narrow range of activities that do not, or rarely, spill over into private households and other domiciled settings” [9,10]. In essence, anchored personal relationships are relationships highly embedded within a social context and exist solely in a shared time, place, and activity. In urban settings, such relationships and interactions are common [11,12] but have not been adequately explored using quantitative approaches. This is partly because these anchored personal relationships are difficult for researchers and respondents to measure. The following section outlines concepts related to anchored personal relationships and summarizes previous research in this area. Subsequently, these findings were used to predict how dog ownership facilitates incidental interactions, friendships, and anchored personal relationships. A subsequent section will discuss the potential for personal relationships fostered by dog ownership, particularly anchored personal relationships, to nurture a sense of community.

### Anchored Personal Relationships

This study was particularly interested in demonstrating that dog ownership fosters the development of anchored personal relationships in addition to incidental interactions and friendships. The relationships among bar patrons provide a simple and accessible way to illustrate the concept of anchored personal relationships. Bar patrons recognize each other and behave amicably only during their time at the bar. Some patrons interact repeatedly, and it is expected that returning to the bar will result in contact with these individuals. However, while they appear intimate within the social context or physical location of the bar, their personal relationships are anchored to the social context and cannot, or can rarely, be decontextualized. It would not be acceptable for bar patrons to invite each other into their homes or to meet elsewhere.

Incidental interactions differ from the interactions that occur in anchored personal relationships. In anchored relationships, participants can expect to encounter a person when they visit a particular social context, such as a bar. Their interactions with this person are not incidental. A further distinction between the two is the level of intimacy among participants and the continuity of their relationships. While anchored personal relationships are confined to a specific context, they have the capacity to foster intimate and enduring connections among participants. There is less capacity for intimacy and connection to develop from incidental interactions.

The contextual nature of anchored personal relationships distinguishes them from friendships. Once formed, friendships do not necessarily require any specific context or interaction to be maintained, and intimacy is a fundamental aspect of these relationships [13]. Although intimacy may develop from anchored personal relationships to a certain extent, individuals do not anticipate receiving social support through these contextualized relationships.

In this study, we focused on anchored personal relationships because we believe that they exemplify the kinds of personal relationships fostered by dog ownership. When walking their dogs, many dog owners follow a specific route and spend some period of time in a park, dog run, or other dog-friendly places. This increases the likelihood of encountering others who also visit these places. Regular visits to the same locations tend to lead to repeated contact, and people begin to recognize each other. Furthermore, the presence of dogs in these spaces fosters interactions among individuals with no pre-existing relationships, including interactions among strangers [6], due to a shared social context. The likelihood of contact is higher when both parties are dog walkers.

Relationships formed in these specific social contexts may serve certain support functions, such as exchanging useful information. However, previous research has suggested that relationships formed through dog ownership do not necessarily develop into decontextualized, intimate relationships such as friendships that extend to other contexts. Communication among dog owners who meet while dog walking is likely limited to information about their dogs [14–16]. A survey of a small sample of dog owners in Japan [17] also found that, although dog ownership increases the number of perceived others with whom they sometimes greet and talk, their conversation topics tend to be limited to dogs. Respondents reported knowing an average of 30.3 names of other individuals’ dogs, but only an average of 11.1 names of the dogs’ owners. Kaneko [16] also found that while Japanese dog or cat owners report having several neighbors with whom they have good relationships, they report the same number of friends as non-owners. These findings suggest that the personal relationships fostered by dogs are dog-centered. Owners tend to have little interest in developing personal relationships with each other, or possibly suppress this interest [14]. The relationships and interactions facilitated by dogs are therefore maintained only within a narrow physical and social context based on shared physical location (e.g., parks) and interest in dogs. It is clear that these relationships have the characteristics of anchored personal relationships.

Anchored personal relationships, such as those fostered by dog ownership, have three characteristics that are expected to have important social consequences. First, unlike incidental interactions, interactions within anchored personal relationships are expected to repeat. This facilitates the development of intimacy. Even if intimacy does not develop, cooperative relationships are likely to form through these repeated interactions [18].

Second, the costs of forming and maintaining anchored personal relationships are low. Interactions within anchored personal relationships are maintained, in part, by sharing social contexts, such as physical places or activities. Individual social ties and the pursuit of social interactions are not always the primary purpose for participating in these contexts. Thus, individuals can interact with those sharing the context without incurring additional social costs. It is also relatively easy to disengage from these anchored personal relationships. For example, it is possible to disengage from anchored personal relationships fostered by dog walking by changing one’s walking route. This is considerably easier than disengaging from relationships with coworkers.

Third, anchored personal relationships are more likely to lead to connections with heterogeneous others than friendships, which are strongly influenced by homophily [19,20]. This may allow individuals to mobilize a greater variety of resources compared to those obtained from friendships [21–24].

Previous studies conducted in Australia and the United States [4,5,7,8] suggest that dog ownership increases the likelihood of incidental interactions and friends in one’s neighborhood. Despite the potential for relationships between neighbors to manifest certain qualities of anchored personal relationships, extant quantitative studies on dog owners’ personal relationships have not yet distinctly addressed anchored personal relationships. This omission may have led to an underestimation of the impact of dog ownership on the development of personal relationships. Therefore, this study employed quantitative methods to examine the associations between dog ownership and incidental interactions, friendships, and anchored personal relationships.

### Anchored personal relationships and sense of community

This study also addressed the association between anchored personal relationships and sense of community. A sense of community is defined as the feeling of having a psychological bond with the community one belongs to. This concept is theorized to comprise four factors: membership, influence, integration and fulfillment of needs, and shared emotional connection [25]. At the individual level, a sense of community is a component of having a sense of belonging which is a fundamental human need [26,27]. Furthermore, previous research demonstrated the community-level importance of a sense of community, such as promoting community participation [28].

Compared to incidental interactions or friendships, anchored personal relationships fostered by dog ownership are more likely to be perceived as ties with neighbors. Individuals rarely travel to areas distant from their residence for the purpose of routine dog-walking. A Japanese study found that the median dog-walking distance is 1.77 km, while the average distance is 2.40 km [29]. Therefore, individuals who repeatedly interact with one another while walking their dogs are likely to be residents of the same neighborhood. Amicable interactions with these individuals may foster trust among neighbors and create a sense of community. This effect is expected to be greater than that of incidental interaction, where interaction partners are less certain that they are residents of the same neighborhood. Furthermore, anchored personal relationships facilitated by dog walking may have a more significant impact on sense of community than friendships. This is because relationships with friends are decontextualized, and regional proximity may not be a salient factor when forming friendships.

### Hypotheses

**Fig 1:**
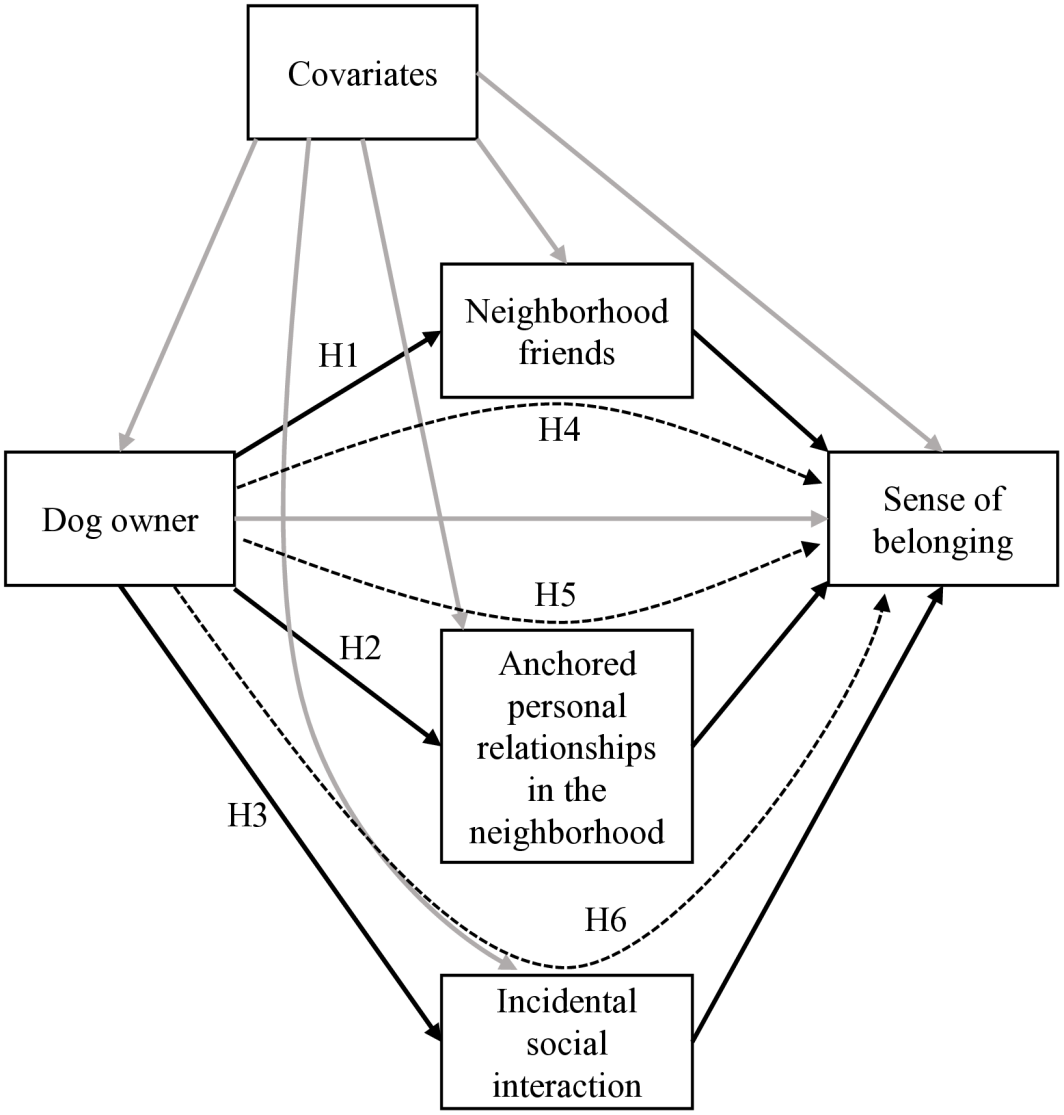
The hypothetical path diagram. Dotted lines denote indirect effects.

Corresponding with the above discussion, this study tested six hypotheses concerning friendships, anchored personal relationships, and incidental interactions. Hypotheses 1, 2, and 3 predicted associations between the three types of personal relationships and dog ownership. Hypotheses 4, 5, and 6 predicted the association between dog ownership and sense of community, as well as the mediating roles played by the three types of personal relationships. A hypothetical path diagram illustrating the hypothesized associations is shown in Fig 1.

Hypothesis 1: Dog owners are more likely than non-owners to have friends in their neighborhoods.

Hypothesis 2: Dog owners are more likely than non-owners to have anchored personal relationships.

Hypothesis 3: Dog owners experience a higher frequency of incidental interactions than non-owners do.

Hypothesis 4: The effect of dog ownership on sense of community is mediated by friendships.

Hypothesis 5: The effect of dog ownership on sense of community is mediated by anchored personal relationships.

Hypothesis 6: The effect of dog ownership on a sense of community is mediated by incidental interactions.

It is important to note that a previous Japanese study found no difference in the number of friends between pet owners and non-owners [16]. Although the Japanese study utilized different measures of friendship compared with existing studies conducted in Australia and the United States, it is plausible that no association between dog ownership and friendship will be observed in the present study, which employs a sample of Japanese participants.

It is also noteworthy that friends and incidental interaction partners may not be perceived as neighbors. Consequently, among the three types of relationships, we expected the magnitude of the mediating effect of anchored personal relationships to exceed that of friendships and incidental interactions.

## Methods

### Ethics statement

This study adhered to the principles outlined in the Declaration of Helsinki as adopted by the World Medical Association. This study was approved by the Ethical Committee for Medical and Health Research Involving Human Subjects of Azabu University (#2490). Respondents did not complete a written consent form because all the data collection and analysis processes were conducted anonymously.

### Participants

Data from a survey conducted in the Central Ward (Chuo-ku, in Japanese) of Sagamihara City in Kanagawa Prefecture were used. The respondents were recruited using convenience sampling and were invited to participate in the academic survey through posters and flyers. Participation was voluntary. The total number of respondents was 517, including 159 men, 351 women, one individual who identified with neither gender, and six individuals who declined answering questions about their gender. Most respondents (approximately 80%) resided in the Central Ward, which has a high density of independent houses and small buildings. There are a small number of parks, green spaces, and water bodies. The remaining respondents were from other wards within Sagamihara City or other subareas within the Tokyo metropolitan area.

### Procedure

The survey was administered in collaboration with the public office of the Central Ward of Sagamihara City, in accordance with a comprehensive cooperative agreement between Azabu University and Sagamihara City. The survey was also conducted in cooperation with local businesses in the shopping district. Advertisements in the form of posters were placed on bulletin boards under the jurisdiction of the Central Ward office. The posters expressed an invitation to participate in a survey titled “The survey of neighborhood connections and well-being.” The poster indicated that the survey objective was to examine the association between dog ownership and two specific factors: 1) social connections, such as bonds with family or the neighborhood; and 2) happiness. The survey instrument, an online questionnaire, was accessible through a QR code printed on the poster. Additionally, flyers sharing the same information as the poster were disseminated by the local government to all nine neighborhood associations in the Central Ward. The flyers were also disseminated through two dog owners’ groups registered with the local government as crime prevention patrol groups. Additionally, the authors of this study disseminated flyers during local community events. During these events, a printed version of the questionnaire was provided to respondents who opted to complete the paper form instead. The completed questionnaires were then collected at the event locations. Staff members of Azabu University residing in Sagamihara City were invited to participate in the survey via e-mail. If they were willing to participate, staff members accessed the online questionnaire using the same details provided on the posters and flyers. All responses within the survey period were considered. Responses from individuals residing outside the Central Ward were not excluded because it was not anticipated that a representative sample of Central Ward residents would be obtained using this method. The survey response rate could not be determined because it was not possible to know how many individuals received flyers, noticed posters, or accessed the questionnaire. The survey was conducted between June 1 and August 31, 2023.

### Indices

#### Pet ownership.

Respondents were asked whether they currently own pets. If they were pet owners, they were asked to specify the type(s) of animal(s) they owned through an open-ended response. They were asked to list all of their pets if they had more than one. Open-ended responses were then coded for dog ownership as “owners” = 1 and “non-owners” = 0 before the analyses were conducted. For control purposes, cat ownership and the ownership of other pets were also respectively coded as “owners” = 1 and “non-owners” = 0..

### Sense of community

Sense of community was measured using a six-item scale developed by Williams and Vaske [25]. Some example items include, “I feel this area is a part of me” and “I am very attached to this area.” The term “this area” was defined a priori as “an area within 30 min of one’s home.” The 30-minute range was chosen to correspond with the methodology of previous studies conducted in Japan. Studies using this method have found that most intimate non-relative relationships among Japanese people fall within this 30-minute range [30]. The same definition was used to measure anchored personal relationships and friendships in the neighborhood when items made use of the term “area”. The items were assessed according to the following response options: “1. very frequently”; “2. frequently”; “3. sometimes”; “4. rarely”; and “5. not at all.” Items were reverse scored as appropriate and subsequently averaged to generate a composite score. This scale had good reliability (α = .82).

### Incidental interactions

Regarding incidental interactions with strangers or acquaintances, we extracted two items from the five-item Minimal Social Interaction Scale [31]. These items assessed the frequency of specific outdoor interactions that would occur during a dog walk: 1) Someone talks to you or asks you questions when they pass by or happen to be present near you, and 2) Vice versa, you talk to them or ask them questions. The response options were “1. often”; “2. sometimes”; “3. rarely”; and “4. never.” Once again, items were reverse scored as appropriate and subsequently averaged to generate a composite score. This scale had moderate reliability (α = .72).

### Anchored personal relationships

To measure anchored personal relationships, we asked respondents, “Sometimes there are individuals who see each other repeatedly in the same places, such as local stores, parks, and public facilities, and recognize each other when they see each other. Do you know of any such individuals?” If the answer was “yes,” the number of such individuals was recorded. This count was not included in the analyses because the distribution was highly skewed to the right, making mediation analyses difficult. Instead, a dummy variable was created to indicate the presence (1) or absence (0) of anchored personal relationships.

### Friendships

We asked respondents whether they had friends who lived in their residential areas, and if so, how many they had. As before, we created a dummy variable for the presence (1) or absence (0) of friends in the analysis.

### Covariates

The associations between dog ownership and well-being indicators have been reported to diminish or disappear when respondent characteristics such as demographics are controlled for [32–35]. It is plausible that the effects of dog ownership on personal relationships, sense of community, and the association between them could disappear after controlling for demographics. Gender, age, educational level, household income, and housing type were used as covariates.

Gender was measured using four categories: male, female, other or neither, and refusal to answer. Seven respondents who answered “other or neither” or declined to answer were excluded from the analysis. Age was calculated based on the respondents’ years of birth. One respondent who reported a birth year of 2022 was excluded from the analysis. Educational level was measured by asking respondents about their highest completed educational level and converting it into years of education. The choices (and years of education after conversion) were elementary and junior high school (9 years), high school (12 years), vocational school (14 years), junior college (14 years), technical college (14 years), university (16 years), and graduate school (18 years). Household income was measured on a seven-point scale. Class values were assigned to different household income categories: “less than 2 million JPY” = 1; “from 2 million to 4 million JPY” = 3; “from 4 million to 6 million JPY” = 5; “from 6 million to 8 million JPY” = 7; “from 8 million to 10 million JPY” = 9; “from 10 million to 12 million JPY” = 11; and “greater than 12 million JPY” = 12. Housing type was measured using five categories: owner-occupied apartments, rented apartments, owner-occupied houses, rented houses, and rented rooms. For the analysis, we dichotomized the data according to whether respondents lived in a house (the third and fourth categories) or an apartment complex (the other three categories). A previous study conducted in the United States [36] suggested that individuals living in independent houses were more likely to own dogs than those residing in apartments. Consequently, we assumed that housing type may be associated with the rate of dog ownership as well as the effects of dog ownership in Japan.

### Analytical strategy

The target variable for the analysis was sense of community. The explanatory variable was dog ownership. The mediating variables included friendships, anchored personal relationships, and the frequency of incidental interactions. Cat and other pet ownership, gender, age, educational level, household income, and housing type were used as covariates. Cat and other pet ownership, gender, and housing type were treated as dummy variables. Respondents with missing values for these were excluded listwise, resulting in the inclusion of 377 respondents.

Given that the mediating variables were dummy coded, generalized structural equation modeling (GSEM) was used. A Gaussian family and identity link function were specified for sense of community and incidental interaction, while a binomial family and logit link were specified for friendships and anchored personal relationships. Incidental interactions, and sense of community were standardized when included in the GSEM.

The *gsem* command in Stata Version 17 was used for analysis. The *bootstrap* commands (2000 iterations) were used to calculate the mediation effects. The seed was set to 12345 using the *set seed* command to ensure replicability of the bootstrap results.

## Results

**Fig 2:**
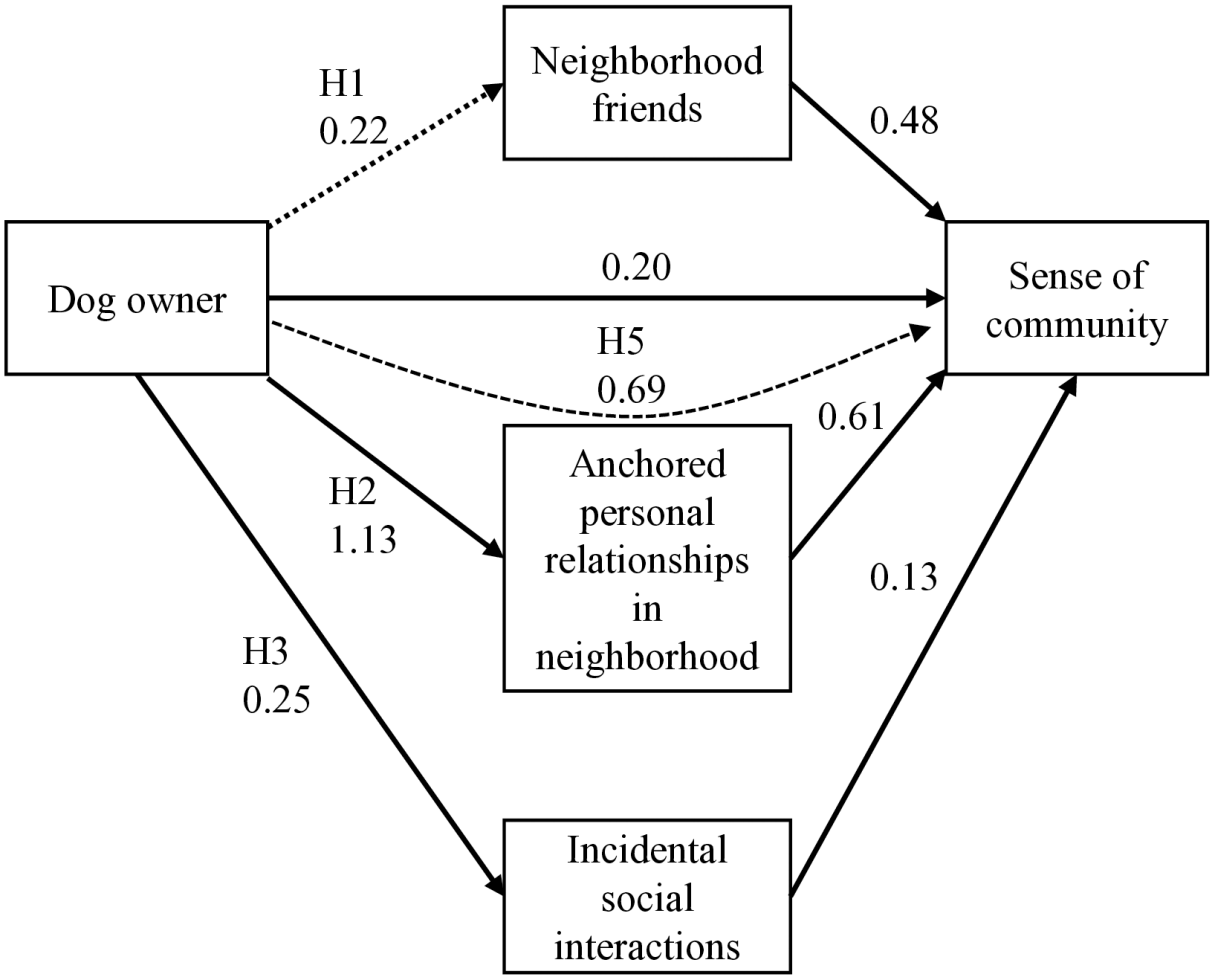
The result of the GSEM. Dotted lines denote indirect effects. Covariates and non-significant paths were omitted.

Descriptive statistics for the variables are presented in Table 1. The GSEM results are shown in Fig 2 and Table 2. As shown on the left side of Fig 2 and in Models (1), (2), and (3) of Table 2, slopes of dog ownership were significant for anchored personal relationships (*b* = 1.13, 95% CI = [0.59, 1.67], *p* < .001) and incidental interactions (*b* = 0.25, 95% CI = [0.04, 0.46], *p* = .019). However, this slope was not significant for friendships (*b* = 0.22, 95% CI = [−0.25, 0.70], *p* = .36). These results support Hypotheses 2 and 3, but not Hypothesis 1. When covariates were not included, the slope of dog ownership was significant for friendships (*b* = 0.49, 95% CI = [0.06, 0.92], *p* = .027). Thus, dog ownership is associated with the likelihood of having friends in one’s neighborhood, but this association may be spurious because of common factors.

**Table 1:** Descriptive statistics of variables.

Variables		Mean	SD	
Incidental social interactions score		2.45	0.80	
Sense of community score		3.35	0.83	
Age (years)		45.63	16.21	
Years of education		14.48	2.24	
Annual household income (in 1 million JPY)		6.12	3.29	
		Proportion		
Friendships (1 or more)		63.40%		
Anchored personal relationships (1 or more)		70.56%		
Owning dogs		42.97%		
Owning cats		10.88%		
Owning other pets		11.41%		
Gender				
Male		33.95%		
Female		66.05%		
Housing type				
Apartment		50.13%		
House		49.87%		
Household income				
Less than 2 million yen	16.98%			
From 2 million to 4 million yen	18.04%			
From 4 million to 6 million yen	16.18%			
From 6 million to 8 million yen	17.77%			
From 8 million to 10 million yen	15.12%			
From 10 million to 12 million yen	8.75%			
Greater than 12 million yen	7.16%			
n = 377				

**Table 2:** Summary of the results of the generalized structural equation modeling.

	Target variables															
	(1)				(2)				(3)				(4)			
	Friendships				Anchored personal relationships				*Incidental social interaction*				Sense of community			
			95% confidence intervals				95% confidence intervals				95% confidence intervals				95% confidence intervals	
Explanatory variables	Slopes	p values	Lower	Upper	Slopes	p values	Lower	Upper	Slopes	p values	Lower	Upper	Slopes	p values	Lower	Upper
Friendships (present = 1)													0.48	0.000	0.31	0.66
Anchored personal relationships (present = 1)													0.61	0.000	0.41	0.80
*Incidental social interaction*													0.13	0.002	0.05	0.22
Dog ownership	0.22	0.357	−0.25	0.70	1.13	0.000	0.59	1.67	0.25	0.019	0.04	0.46	0.20	0.028	0.02	0.38
Cat ownership	0.02	0.962	−0.71	0.75	0.16	0.709	−0.68	1.00	0.09	0.593	−0.23	0.41	0.21	0.129	−0.06	0.47
Other pet ownership	0.10	0.786	−0.61	0.81	0.69	0.107	−0.15	1.53	−0.06	0.695	−0.38	0.25	0.09	0.519	−0.17	0.34
*Age*	0.00	0.980	−0.01	0.01	0.02	0.013	0.00	0.03	0.01	0.003	0.00	0.02	0.01	0.000	0.01	0.02
Female	0.85	0.000	0.38	1.33	0.17	0.519	−0.35	0.70	0.32	0.004	0.10	0.53	−0.27	0.004	−0.45	−0.08
Years of education	0.00	0.999	−0.11	0.11	0.01	0.881	−0.11	0.13	−0.03	0.193	−0.08	0.02	−0.01	0.568	−0.05	0.03
Annual household income	−0.03	0.361	−0.11	0.04	0.10	0.017	0.02	0.18	0.04	0.032	0.00	0.07	0.01	0.340	−0.01	0.04
Housing type (House = 1, Apartment = 0)	0.95	0.000	0.47	1.43	0.57	0.031	0.05	1.10	0.12	0.252	−0.09	0.34	0.12	0.177	−0.06	0.30
Intercept	−0.34	0.704	−2.08	1.40	−1.56	0.112	−3.50	0.37	−0.66	0.093	−1.43	0.11	−1.27	0.000	−1.92	−0.63
n = 377																
Italics denote variables are standardized.																

As shown in the right part of Fig 2 and Model (4) in Table 2, the presence of friends (*b* = 0.48, 95% CI = [0.31, 0.66], *p* < .001), anchored personal relationships (*b* = 0.61, 95% CI = [0.41, 0.81], *p* < .001), and incidental interactions (*b* = 0.13, 95% CI = [0.05, 0.22], *p* = .002) in the neighborhood were all positively associated with sense of community. The direct effect of dog ownership (*b* = 0.20, 95% CI = [0.02, 0.38], *p* = .028) was also significant. This finding was robust to removing the covariates.

The indirect effect of dog ownership on sense of community was significant only through anchored personal relationships (*b* = 0.69, 95% CI = [0.29, 1.08], *p* < .001). The indirect effects of friends (*b* = 0.11, 95% CI = [−0.14, 0.35], *p* = .39) and incidental interactions (*b* = 0.03, 95% CI = [−0.01, 0.07], *p* = .108) in the neighborhood were not significant. Thus, the results supported Hypothesis 5, but not Hypotheses 4 and 6. Given that the direct effect of dog ownership was significant, the mediating effect of anchored personal relationships was partial.

When the covariates were removed from the analysis, the indirect effects of friends (*b* = 0.22, 95% CI = [0.00, 0.43], *p* = .045) and incidental interactions (*b* = 0.06, 95% CI = [0.01, 0.11], *p* = .019) in the neighborhood became significant, suggesting spurious indirect associations between dog ownership and sense of community through these variables. In all the analyses described above, no slopes for cat ownership or ownership of other pets were significant.

## Discussion

### Testing the hypotheses

This study examined the contribution of dog ownership to the formation of personal relationships and the resulting changes in sense of community. Social survey data were analyzed using GSEM, and the results indicated that dog owners were more likely to have anchored personal relationships and experienced more incidental interactions. These findings support Hypotheses 2 and 3. However, the likelihood of having friends in the neighborhood did not change with dog ownership when controlling for demographic variables. This finding is consistent with a previous Japanese study [16], although it differs from findings by Wood et al. in Australia and the United States [5]. Methodological discrepancies may be responsible for these divergent results. Wood et al. directly asked their respondents whether they have “at least one person met through pet as a friend”, whereas the present study asked respondents whether they have friends in the neighborhood, irrespective of whether the respondents’ dogs played a role in these relationships. Another explanation is that this study controlled for demographic factors. Further research is needed to determine whether dog ownership increases the likelihood of making friends in one’s neighborhood, while controlling for demographic variables and other characteristics of dog owners, such as personality.

Friends, anchored personal relationships, and incidental interactions in the neighborhood were all positively associated with sense of community; however, only anchored personal relationships significantly mediated the effects of dog ownership. Thus, Hypothesis 5 was supported, but Hypotheses 4 and 6 were not. Notably, the mediating effect of anchored personal relationships was more than twice as large as the direct effect of dog ownership on sense of community. This suggests that anchored personal relationships play an important role in fostering a sense of community through dog ownership. One of the central points of this study was the need to consider anchored personal relationships as relationships fostered by dog ownership, and the results support this claim. Thus, future research should consider personal relationships embedded in specific social contexts and shared interest in dogs, in addition to incidental interactions and friendships.

### Other pets and personal relationships

In this study, respondents were asked to report all pets that they owned. We coded the open-ended responses for ownership of three types of pets: dogs, cats, and other pets. Cat and other pet ownership were controlled for as a covariate in the model. However, as shown in Table 2, ownership of cats and other pets was not significantly associated with the three types of personal relationships or sense of community. Previous studies have reported that dog ownership has more consistent and stronger associations with personal relationships [5], mortality [37], and subjective well-being [38] than ownership of other pets. The results of this study are consistent with these findings. However, it should be noted that this study predicted dog ownership to increase opportunities to experience incidental interactions and develop anchored personal relationships because pet dogs require regular outdoor walks. Notably, other personal relationships such as those existing outside one’s residential area may not have been captured by the measures used in this study. For example, owners of the same type of pet may develop intimate relationships based on this shared interest, and these relationships may be independent of physical distance.

### Limitations and future directions

Although this study contributes to literature on pet ownership and interpersonal relationships, several limitations and unresolved issues must be addressed in future research. First, this study predicted that dog ownership would enhance human-human interactions, primarily because dogs require regular walks. However, owing to data constraints, we were unable to differentiate between dog owners who regularly engaged in dog walking and those who did not. If dog ownership, independent of walking habits, affects personal relationships, it would arise from a different process than hypothesized in this study. Future studies should differentiate between the effects of dog ownership and dog walking habits. In addition, future studies should consider the frequency, duration, and distance of dog walks. Furthermore, the consideration of dog characteristics may facilitate the disentanglement of the association between dog ownership and personal relationships. For instance, previous studies have demonstrated that pet dogs displaying problematic behaviors prevent owners from interacting with other pet owners during dog walking [39–41]. Consequently, owning dogs exhibiting problematic behavior may not contribute to the formation and maintenance of personal relationships.

Furthermore, it would be beneficial to examine which types of places visited with dogs correspond with larger increases in personal relationships and social interactions. This contributes to the ongoing discussion of pet dogs’ access to public spaces. Similarly, a comparative analysis of the effects of dog ownership and ownership of other types of pets would be worthwhile. If walking habits are crucial to the formation and maintenance of personal relationships, individuals who do not walk their dogs should have a similar number of personal relationships as non-owners and owners of other pets. Conversely, according to the homophily principle, which posits that individuals tend to form relationships with similar others [20], owning the same type of pet may facilitate the social ties between owners. However, this study is limited to neighborhoods, precluding the measurement of such relationships. A broader measurement framework encompassing online ties is imperative for comprehensively assessing the impact of personal relationships. To the best of our knowledge, no previous study has quantitatively compared the personal relationships of owners of various types of pets.

Second, this study relied on data obtained from a cross-sectional survey. Consequently, it was not possible to confirm a causal relationship between dog ownership, personal relationships, and sense of community. The most plausible alternative explanation for the correlation between the variables is the existence of common factors. Although several demographic factors were controlled in this study, we did not control for personality and built environment characteristics. For example, some dimensions of the Big Five personality traits [42] may increase or decrease the likelihood of both pet ownership and interactions with strangers and acquaintances. Previous studies have indicated that the number of social ties is associated with extraversion and neuroticism. This association is more pronounced when considering ties with non-intimate individuals than those with intimate ones [43–45]. Concurrently, studies have found a positive association between dog and cat ownership and extraversion [46,47]. This suggests that the association between dog ownership and personal relationships, particularly between anchored personal relationships and incidental interactions, may be inflated. The association between anchored personal relationships or incidental interactions and sense of community is likely causal in both directions. Longitudinal studies and experimental treatments are needed to investigate this association. Several studies have already examined the association between pet ownership and indicators of well-being using such methods.

Third, the sample characteristics were not fully considered. Dog owners may have been disproportionally included in the sample of this study. The study’s objective was to explore the associations between dog ownership and personal relationships and this was disclosed to the respondents before the survey was administered. While approximately 40% of the respondents in this study reported owning dogs, a recent large-scale Japanese online survey revealed that the dog ownership rate was only 9.10% in 2023 [48]. It is also plausible that individuals with a strong sense of community were more inclined to participate in the survey because a proportion of the respondents were recruited at local community events or through community organizations. Cultural factors were not considered in the sample characteristics. A previous study [49] suggests that the experience of minimal social interaction, a concept similar to incidental interaction, is correlated with relational mobility [50]. Most social and cultural psychology studies have suggested that relational mobility is low in Japanese society [51]. Similarly, the results of an international comparative survey suggested that Japanese people are reluctant to interact with strangers; only 21% of respondents, the lowest in the world, reported having helped a stranger within the last month [52]. This may be one reason that dog ownership did not correlate with having friends in the Japanese context. Support functions offered by incidental interactions and anchored personal relationships may also be weak in this cultural context.

Fourth, geographical and built environmental conditions should be considered. Although the study included a diverse sample of Japanese people, it was not representative, with the majority of respondents residing in a suburb of a Japanese metropolitan area. Most respondents resided in the Central Ward of Sagamihara City, a locale characterized by a high density of independent houses and small buildings, with a paucity of parks, green spaces, and water bodies. Consequently, there may have been a lack of suitable locations for dog walking, which may have led to an increase in anchored personal relationships since dog walkers are concentrated in a few specific locations. If this conjecture is true, the association between dog ownership and anchored personal relationships observed in this study may be exaggerated. Future studies should aim to replicate the findings of this study with consideration of geographical and built environment conditions.

Fifth, the impacts of having a strong sense of community were not addressed. Previous studies have suggested that sense of community is positively correlated with subjective well-being [27,53]. The indirect effects of dog ownership through sense of community are worth exploring, as the relationships between dog ownership and well-being indicators in the general population are inconsistent. Indirect relationships between dog ownership and prosocial behavior should also be explored, as a sense of belonging is positively correlated with prosocial behavior [54,55]. If dog ownership increases prosocial behavior, then the presence of pet dogs may have a positive impact on the communities where their owners reside. Dog ownership, especially in urban areas, has negative spillover effects, including but not limited to fecal- and noise-related issues. It is important to note that the personal benefits enjoyed by dog owners do not justify the presence of dogs in the community. By examining the contributions of pet dogs at the community level, it may be possible to assert the societal benefits of the presence of pet dogs within the community. Research has already been conducted on dog walking for crime prevention [56], and it is possible that the presence of dogs makes further contributions, such as increasing participation in community activities.

## Conclusion

Despite several limitations, this study demonstrated the potential of dog ownership to cultivate a sense of community through the development of personal relationships. It showed that anchored personal relationships play an important role in this indirect effect. Anchored personal relationships were the type of personal relationship that was most strongly correlated with dog ownership and sense of community; however, this association has rarely been addressed in previous quantitative studies. Future studies should include anchored personal relationships to disentangle the effects of dog ownership at the individual, social, and societal levels.
